# Seroprevalence of West Nile, Usutu and tick-borne encephalitis viruses in equids from south-western France in 2023

**DOI:** 10.1186/s13567-025-01508-w

**Published:** 2025-04-24

**Authors:** Noémie Chevalier, Camille V. Migné, Teheipuaura Mariteragi-Helle, Marine Dumarest, Margaux De Mas, Manon Chevrier, Emilie Queré, Christel Marcillaud-Pitel, Coralie Lupo, Clément Bigeard, Thierry Touzet, Agnès Leblond, Benoît Durand, Marianne Depecker, Gaëlle Gonzalez

**Affiliations:** 1 Clinique de Conques, Centre Hospitalier Vétérinaire Équin, 33420 Saint-Aubin-de-Branne, France; 2 UMR Virologie, Laboratoire de Santé Animale, ANSES, INRAE, École Nationale Vétérinaire d’Alfort, 94700 Maisons-Alfort, France; 3 École Nationale Vétérinaire de Toulouse, 31300 Toulouse, France; 4 Écolecole Nationale Vétérinaire de Maisons-Alfort, 94700 Maisons-Alfort, France; 5 RESPE (French Network for the Surveillance of Equine Diseases), 14280 Saint-Contest, France; 6 Direction Départementale de La Protection des Populations de Gironde, 33520 Bruges, France; 7 UMR EPIA (Épidémiologiepidémiologie des Maladies Animales et Zoonotiques), INRAE, Université de Lyon, VetAgro Sup, 69280 Marcy L’Etoile, France; 8 EPIMIM, Laboratoire de Santé Animale, Anses, École Nationale Vétérinaire d’Alfort, 94700 Maisons-Alfort, France

**Keywords:** West Nile virus, Usutu virus, tick-borne encephalitis virus, seroprevalence, horse

## Abstract

The circulation of West Nile virus (WNV), Usutu virus (USUV), and tick-borne encephalitis virus (TBEV) was investigated in south-western France during the first six months of 2023, following the emergence of WNV in equids in Gironde, a département in south-western France, in 2022. Blood samples were collected from 494 horses located in the Gironde département and divided into three zones: the Confluence zone, the Intermediate zone and the Arcachon Basin. Samples were tested for WNV-, USUV- and TBEV-specific antibodies. An overall seroprevalence of 14% (95% CI [11–18%]) for orthoflavivirus antibodies was detected in Gironde. The highest seroprevalence rates for WNV and USUV were observed in the Confluence Zone (9%, 95% CI [6–13%] and 5%, 95% CI [3–8%], respectively), where the type of housing (animals kept in pasture only) and proximity to a special bird protection area were identified as risk factors for WNV seropositivity. This study presents the first seroprevalence investigation of WNV, USUV and TBEV infections in equids located on the Atlantic coast of France and demonstrates intense circulation of WNV in this region, as well as evidence of equine USUV-specific infection.

**Supplementary Information:**

The online version contains supplementary material available at 10.1186/s13567-025-01508-w.

## Introduction

West Nile virus (WNV) is a reemerging zoonotic mosquito-borne orthoflavivirus (*Flaviviridae* family) that represents a growing threat to human and animal health. It is one of the main viral agents causing encephalitis in humans and horses in Europe [1]. It was identified for the first time in Uganda in 1937 [2], and its circulation is now evident on all five continents [3]. WNV is maintained in an enzootic cycle involving ornithophilic mosquitoes as vectors (*Culex* spp.) and avian reservoirs and/or amplifying hosts. The virus can infect approximately one hundred mammalian host species, which are considered epidemiologically dead-end hosts that do not participate in virus spread [4]. Among them, humans and horses are known to develop symptoms following WNV infection. WNV infection is predominantly asymptomatic or can cause flu-like syndrome. In 1 to 10% of cases, neurological signs, the most common of which are ataxia, weakness and muscle fasciculations, are encountered. Some horses develop signs of encephalitis, leading to death or euthanasia [5–7]. In addition, human-to-human transmission of WNV during blood transfusions or organ transplants has been extensively documented and remains a major concern in the transfusion context [8, 9]. To reduce the risk of nonvector transmission, Europe introduced precautionary measures to control labile products, such as the exclusion of blood donors for 28 days after a visit to a high-risk area (European Directive 2004/33/EC) [9].

As WNV circulation is heterogeneous between Member States and is a compulsory notifiable disease for birds (since 2021), equines and humans (since 2009) to the European Commission [10], each country has implemented an appropriate surveillance and monitoring system to detect WNV cases in Equidae, avifauna and humans.

Over the last decade, Europe has faced significant changes in the distribution of WNV, as well as two other mosquito-borne orthoflaviviruses, Usutu virus (USUV) and tick-borne encephalitis virus (TBEV), most likely caused by global change and increasing global warming. The three viruses actively cocirculate in most European countries. Infections are mainly subclinical, and accurate estimations of infection incidence rates are difficult.

The surveillance of WNV infection relies heavily on neurological syndromic surveillance in humans and horses in most EU countries. Serosurveillance of equines is often employed to detect virus circulation in a territory and allows estimation of the intensity of circulation in that territory [3, 11–17]. The outdoor lifestyle of equines and their proximity to humans are good indicators of WNV circulation and human exposure.

**Figure 1:**
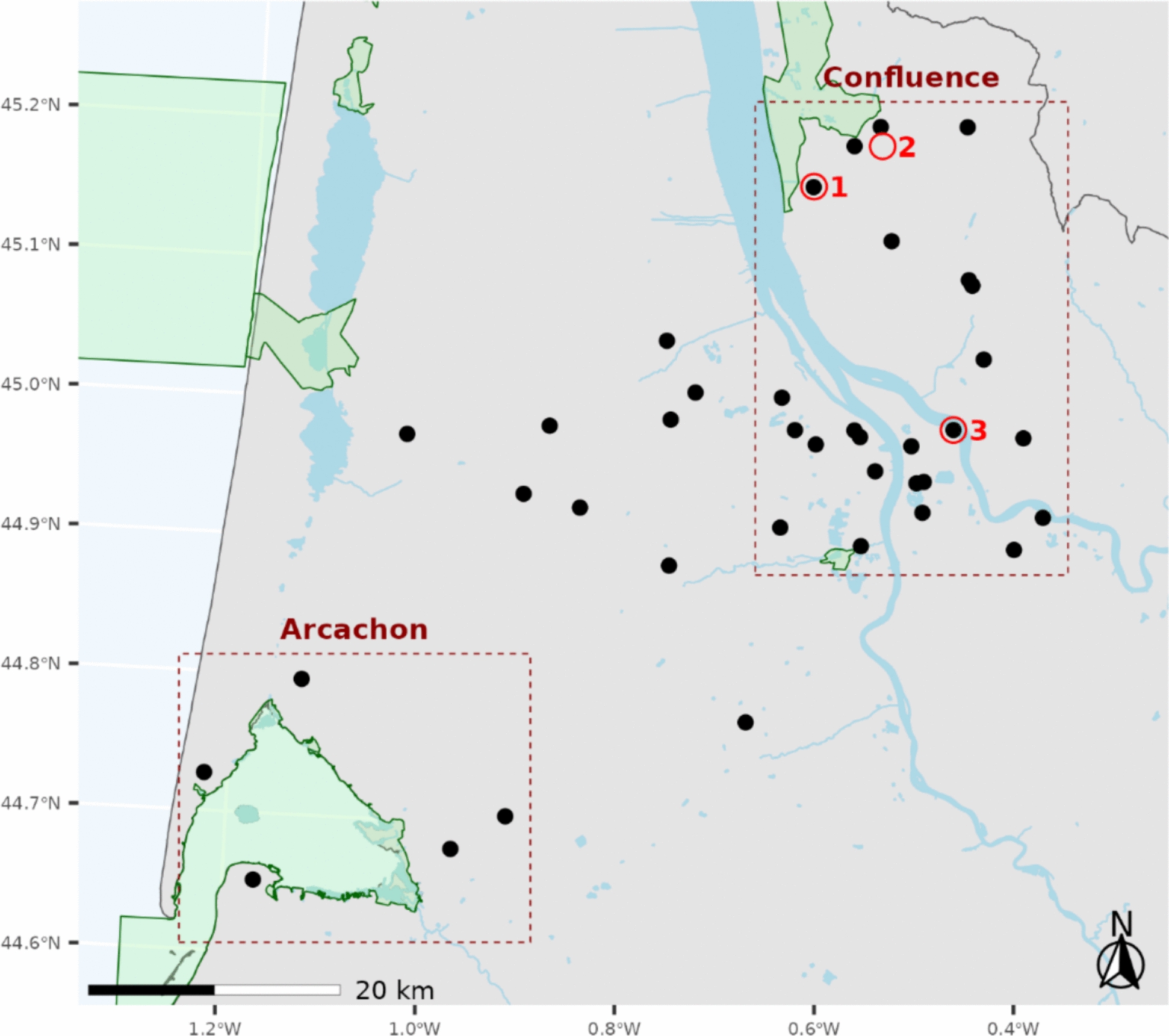
**Map of the study area in south-western France, with black dots representing the stables where horses were sampled, grouped into two main zones: the Confluence and Arcachon zones.** The red circles indicate the stables where clinical cases were reported in 2022, and the green polygons indicate the special protection areas for the protection of wild birds.

In France, WNV was detected for the first time in the Camargue region in 1962. Between 2000 and 2021, several WNV outbreaks of varying intensity were reported, all of which were located in the Mediterranean region and Corsica [18–21]. The 2022 transmission season was marked by the emergence of WNV in south-western France, which is located on the Atlantic coast. In October 2022, three horses of three distinct stables in Gironde, a département in this region (Figure 1), exhibited neurological signs, including ataxia, weakness, muscle fasciculations, and encephalitis. Acute WNV infection was confirmed by serological diagnostic methods (ELISA for IgM and IgG) carried out by the French National Reference Laboratory for WNV. USUV was detected for the first time in 2015 in blackbirds in the Rhône and Haut-Rhin départements [22]. A record circulation of the USUV was recorded in 2018, with earlier and more widespread detection than in previous years [23]. In 2022 and 2023, the USUV continued to circulate actively in France, with a human case diagnosed in Nouvelle Aquitaine region [24]. TBEV has been an increasing concern in France in recent years, with a slight increase in the number of cases reported since 2015 [25]. TBEV infection has been a notifiable disease in France since May 2021 [26].

To obtain an accurate assessment of orthoflavivirus circulation in Gironde, the first seroprevalence survey was conducted among equids located near stables where WNV cases in horses were newly reported in 2022 by investigating possible seroconversion to WNV, USUV and TBEV (Figure 1).

We aimed to provide valuable insights into the intensity of WNV circulation in 2022 in this newly emerging West Nile virus zone and the associated risk factors for infection. We also evaluated the occurrence of UUV and TBEV in horses in this area.

## Materials and methods

### Geographical zones

The survey was conducted in Gironde, south-western France, which is characterized by its contrasting landscape. The epidemiological parameters of the three defined zones were determined. The first zone, named Confluence, corresponds to the area where WNV-infected horses were reported in 2022 (Figure 1). The confluence lies at the junction of two rivers, the Garonne River and the Dordogne River, where large populations of mosquitoes can be expected. The second zone corresponds to the Arcachon Basin. The Arcachon Basin lies at the heart of the East Atlantic Flyway, one of the eight most important bird migration corridors on the planet. No cases of WNV infection were reported here [27]. These two zones offer a protected habitat for resident and migratory bird species (represented in green in Figure 1) that use wetlands as a stopover during their long journeys. These avian tranquility zones encourage interactions between local mosquitoes and competent avian hosts, which promotes the establishment of the bird‒mosquito‒bird transmission cycle of WNV and USUV. The third zone covers the area between the Confluence basin and the Arcachon Basin.

### Study design and sample collection

The study was a cross-sectional prospective study. It took place in April and May 2023, prior to the occurrence of seasonal orthoflavivirus activity, to reflect the exposure of horses to WNV, USUV and TBEV during the summer of 2022, as IgG and neutralizing antibodies tend to have a longer duration in infected horse sera [28, 29]. Animals were recruited by the Clinique Equine de Conques (Saint-Aubin-de-Branne, Gironde, France) through social media and client mailing, according to our three different zones. All owners signed a consent form, and the treating veterinarians were informed of the procedure. This study was approved by the Ethics Committee for Clinical Research (ComERC) of the Veterinary School of Alfort (EnVA) (Agreement number: 2023-06-23).

A convenience-sampling scheme that involved participants who were easily accessible and willing to participate in the study was applied. Serum samples were collected from 494 adult (> 2-year-old) client-owned equids residing in 39 different stables of the three studied zones: 306 equids from 25 stables in the Confluence zone, 77 equids from 5 stables in the Arcachon zone, and 111 equids from 9 stables in the Intermediate zone (Figure 1). The animals were unvaccinated against WNV and had been living in the sampling area for the past 6 years throughout 2022 without travelling to the Mediterranean region.

The number of equids sampled was stable in size: all horses were sampled from small stables (1 to 6 animals), 10 horses were sampled from medium stables (between 10 and 15 animals), and 15 horses were sampled from large stables (more than 15 animals). The average and median age of the horses was 12 years. The 25% and 75% percentiles were 7 and 17 years, respectively. All the animals were asymptomatic at the time of sampling. Three cases from 2022 were not included in our cohort, although two of the three corresponding stable cases were included (Figure 1).

Serum samples were collected from each included animal. Blood samples were collected in vacutainer dry tubes and centrifuged at 5000 rpm for 5 min within 24 h of collection. The serum was separated, stored at 4 °C, and sent to the French National Reference Laboratory for West Nile virus, ANSES (Maisons-Alfort, France), for further analysis.

In addition to sample collection, each stable manager/owner completed a survey consisting of personal face-to-face interviews to gather information on individual characteristics and stable management as well as their perceptions of the nuisance caused by mosquitoes. This information included age, sex, hair colour, and type of housing. We also collected information about the environment, such as the distance to the nearest water surface and the distance to the northern special protection areas (SPA) located in the Confluence zone. SPAs have been created for the protection of wild bird species listed in Annex 1 of the EU bird directive (Directive 79/409/EEC) or that serve as breeding, moulting, wintering, or staging areas for migrating birds. Five SPAs have been defined in the study area around wetlands (Figure 1).

### Serological testing

#### ELISA tests

The serum samples were first tested for IgG flaviviruses via the commercial Pan-Flavivirus ELISA “ID Screen Flavivirus competition” (Innovative Diagnostic, Montpellier, France). The protocol was performed according to the manufacturer’s instructions. The results are expressed as %S/N. If a sample had a %S/N less than or equal to 40%, the sample was considered positive. If it is strictly greater than 50%, the sample is negative. If the result is between 40 and 50%, the sample is considered doubtful. Positive and doubtful results were confirmed for the presence of WNV-, USUV- and TBEV-specific neutralizing antibodies using specific virus neutralization tests.

#### Virus-neutralization test (VNT)

To identify specifically against which flaviviruses the IgG antibodies detected by ELISA are directed, a virus neutralization test (VNT) was carried out for the three main orthoflaviviruses circulating in France: WNV, USUV and TBEV. This test was performed in 96-well plates as described in Beck et al. [20].

Briefly, the sera were diluted in a cascade by a factor of 2, from 1:5 to 1:160, in a final volume of 100 µL. Fifty microliters of virus was added at an infectious dose of 100 TCID_50_. After 1h30 of incubation at 37 °C and 5% CO_2_, 100 µL of Vero cells was added at a concentration of 2.10^5^ cells/mL. The neutralizing antibody titre was obtained by observing the cytopathic effect (CPE) under a light microscope after 3, 4 and 5 days of incubation at 37 °C and 5% CO_2_ for WNV, USUV and TBEV, respectively. The last dilution of serum showing no CPE or a proportion of CPE less than a quarter of the well observed was noted as the dilution of serum considered where there was still protection against the virus. The neutralizing antibody titre was therefore the inverse of the serum dilution. A serum sample was considered positive if it displayed protection (no CPE) for a neutralizing dilution ≥ 1:10 for only one orthoflavivirus [30]. Sera neutralizing more than one virus were considered positive for the virus neutralized at a fourfold higher dilution than all other viruses. Sera were identified as those containing antibodies against the virus displaying the highest positive serum dilution. If a fourfold difference in neutralizing antibody titres was not reached, the serum was considered undifferentiated between the viruses. Sera that tested positive by competitive ELISA and negative by VNT were considered negative.

### Statistical analysis

All collected variables were described in terms of frequency distribution (qualitative data) or median and range (quantitative data) classified by serological status.

Although we checked during the survey that the sampled horses had no travel history to the Mediterranean region, we did not have access to their travel history inside the study region. Later, some horses had moved between the three zones. In particular, the horses in the Arcachon zone had been living for several months while the Confluence zone was stable the year preceding the survey. Additionally, the Intermediate zone is a rather dry zone that is covered mainly by forests and is ecologically different from the confluence zone, which is a mosaic of pastures, woods, cultivated areas and vineyards. For this reason, and to minimize the impact of classification errors due to horse movements, we chose to focus on the Confluence zone in the remainder of the statistical analysis. Donkeys were excluded from the analysis because their physiological characteristics differ from those of horses. Data related to the horses and stables of the Confluence zone were analysed using mixed effects logistic regression models, with the stable as a random effect. The dependent variable was the serological status of the animals according to the seroneutralization test. Fixed effects were first individual-level data: animal age (years), sex (male or female), hair colour (light or dark), and type of housing (always on pasture, or both on pasture and indoors). There were 150 water bodies in the confluence zone, mostly segments (*N* = 68) of low-flow natural rivers (as the study region is rather flat, with a maximum altitude of just 100 m and no fast-flowing rivers), water reservoirs (*N* = 50) and gravel pit water bodies (*N* = 25). The others were estuaries, marshes and lakes. *Culex* breeding sites can be found in these habitats, and the distance in kilometres (km) to the nearest water surface was added as a fixed effect.

Two SPAs were present in the Confluence area: a large one to the north and a smaller one to the south (Figure 1). Both were marshland areas. The distances in km to these two SPAs were also added to the list of fixed effects.

Starting from the model with all the fixed effects, we selected the most parsimonious model via backwards model selection on the basis of the likelihood ratio test, *p* value < 0.05. The existence of spatial autocorrelation between observations may bias parameter estimation in statistical models. To determine if this was the case, we tested the spatial autocorrelation of the model deviance residuals [31] via Moran’s I test for increasing distances in the range of the between-stable distances inside the Confluence zone. The exponentiated regression coefficients produced odds ratios (ORs) as a measure of the effect of the variables.

We used the nonparametric approach proposed by [32] to test whether the associations between positive VNT results and WNV, USUV and TBEV (i.e., the number of equids positive for 1, 2 or 3 of these viruses) significantly differed from the expected distribution under the null hypothesis of independent positive results. We also carried out this test at a stable level, considering a stable test to be positive if at least one horse was VNT positive.

All the statistical analyses were performed using R 4.3.3 (R Core Team 2024). Mixed effects logistic regression models were fitted using the lme4 package [33]. Model selection was performed using the buildmer package [34]. Spatial autocorrelation was tested using the pgirmess package [35].

## Results

This study included 494 adults: 334 horses and 160 ponies, 271 of whom were male and 223 of whom were female. The majority (412 horses) were active at the time of sampling: 328 animals lived in pasture, whereas 166 had a mixed lifestyle (stable and pasture). In terms of hair coat colour, 331 animals had a dark coat, whereas 163 had a light coat.

### ELISA and VNT

Among the 494 horses included in the study, 70 were positive for orthoflavivirus antibodies according to competitive ELISA, yielding a seroprevalence rate of 14% (95% CI [11%-18%]). Among them, 60 animals were located in 20 stables in the Confluence zone, 5 horses were housed in 4 stables in the Intermediate zone, and 1 was based on 1 stall in the Arcachon area (Table 1).

**Table 1:** **Comparative serological results among selected horses in the Confluence, Intermediate and Arcachon zones in the Gironde département**

		Geographical zone		
		Confluence	Intermediate	Arcachon
Tested horses/stables		306/25	111/9	77/5
ELISA results				
Positive horses/stables Animal prevalence (95% CI)		64/20 21% (16–26%)	5/4 5% (2–10%)	1/1 1% (0–7%)
VNT results in ELISA-positive animals:				
Positive horses/stables	WNV USUV TBEV	27/13 14/5 6/4	3/2 1/1 1/1	1/1 0/0 0/0
Animal prevalence (95% CI)	WNV USUV TBEV	9% (6–13%) 5% (3–8%) 2% (1–4%)	3% (1–8%) 1% (0–5%) 1% (0–5%)	1% (0–7%) 0% (0–5%) 0% (0–5%)

**Figure 2:**
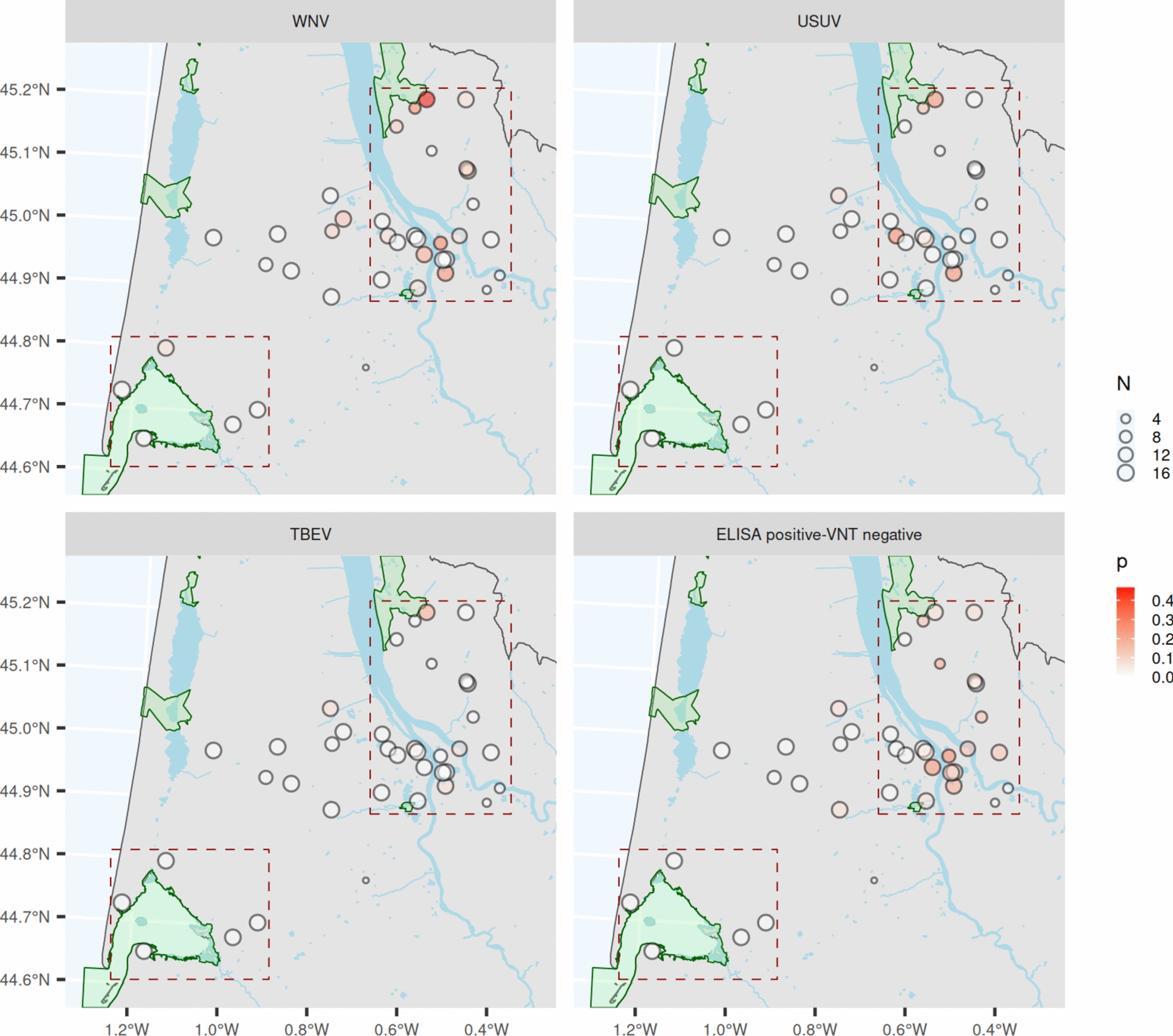
**Distribution of WNV, USUV and/or TBEV seropositive equids in the Confluence zone, the Arcachon Basin and the Intermediate zone in the Gironde département in 2023**. The circles represent the stables selected for the study, the size of which is proportional to the number of sampled horses. The darker the color of the circles is, the greater the number of seropositive horses.

Within the Confluence zone, 27 equids from 13 different stables were positive for WNV by VNT (9%, 95% CI [6–13%]). Within the same zone, 14 equids from 5 different stables were positive for USUV (5%, 95% CI [3–8%]), and 6 equids from 4 different stables were positive for TBEV (2%, 95% CI [1–4%]). In the intermediate zone, 3 equids housed on 2 stables were positive for WNV (3%, 95% CI [1–8%]), one horse was positive for TBEV, and another horse was positive for USUV neutralizing antibodies (1%, 95% CI [0–5%]). For the Arcachon zone, only one horse was positive for WNV (1%, 95% CI [0–7%]), and no horse was seropositive for the other viruses (Table 1 and Figure 2). As the horse had been living for several months in a stable Confluence zone the year preceding the survey, we consider it an artefact.

Undifferentiable VNT results were also observed. In total, 24 equids tested positive for WNV, 5 horses tested positive for both WNV and USUV, 2 horses tested positive for WNV and TBEV, and none tested positive for all three viruses simultaneously (WNV, USUV, TBEV). Additionally, 9 horses tested positive for USUV only, 1 horse tested positive for both USUV and TBEV, and 4 horses tested positive for TBEV only (Table 2).

**Table 2:** **Association between VNT serological status for WNV, USUV and TBEV**

WNV	USUV	TBEV	Number of animals
Positive	Positive	Positive	0
Positive	Positive	Negative	5
Positive	Negative	Positive	2
Positive	Negative	Negative	24
Negative	Positive	Positive	1
Negative	Positive	Negative	9
Negative	Negative	Positive	4
Negative	Negative	Negative	449
Total			494

The individual characteristics of the sampled animals as a function of their WNV and USUV serostatus are shown in Table 3. The qualitative comparison revealed that horses that tested positive for WNV were mainly males kept on pasture only. This does not apply to horses seropositive for USUV.

**Table 3:** **Characteristics of horses as a function of WNV and USUV serostatus**

Individual horse characteristics	WNV		USUV	
	Positive horses	Negative horses	Positive horses	Negative horses
Sex				
Male	19 (70%)	162 (58%)	8 (57%)	173 (59%)
Female	8 (030%)	117 (42%)	6 (43%)	119 (41%)
Age	13 (2–30)	11 (2–35)^a^	10.5 (6–25)^a^	11 (2–35)^a^
Hair colour				
Dark	14 (52%)	197 (71%)	9 (64%)	202 (69%)
Light	13 (48%)	82 (29%)	5 (36%)	90 (31%)
Type of housing				
Pasture only	23 (85%)	158 (57%)	9 (64%)	172 (59%)
Pastures and indoors	4 (15%)	121 (43%)	5 (36%)	120 (41%)

^a^Median and range, in years.

### Risk factor analysis

A mixed effects logistic regression model of VNT serostatus was fitted for WNV and USUV only since, for TBEV, the number of seropositive stables and equids was very low. Furthermore, we focused our analysis on the Confluence zone, where the vast majority of WNV- and USUV-seropositive equids (306 equids) were located (Table 3). For WNV, starting from the full model (i.e., including all the fixed effects, Additional file 1), backwards model selection allowed us to identify two risk factors for VNT seropositivity: the type of housing with a strongly increased risk in animals always kept on pasture (odds ratio [OR] = 3.64) and the distance to the SPA to the north of the Confluence zone (Figure 1), with a decreasing risk when this distance increased ([OR] = 0.93 for a 1 km increase) (Table 4). Other variables (age, sex, hair colour and distance to the nearest water surface) were not retained in the selected model. No spatial autocorrelation was detected in the residuals of either the full model or the model resulting from the selection procedure, suggesting that these models captured the main determinants of spatial variations in risk. For the USUV, model selection resulted in an empty model (containing the intercept only) and did not allow the identification of any seropositivity risk factors.

**Table 4:** **Mixed effects logistic regression model of the VNT WNV serological status of the horses sampled in the Confluence zone**

Variable^a^	Value	*p* value	Odds-ratio ^b^(95% CI)
Intercept		0.002	0.10 (0.03–0.27)
Type of housing	Pasture and indoors		
	Pasture only	0.04	3.64 (1.11–13.15 -17.47)
Distance to the Northern SPA	Increase by 1 km	0.03	93 (87–99)

^a^Reference class for the calculation of the odds ratio.

^b^Confidence interval.

Finally, we detected a significant association between WNV and USUV serological status (*p* = 0.006), with the number of equids positive for both viruses (five of the 494 equids, located in two stables) being significantly greater than the expected 95% interval under the null hypothesis (0–4 animals). Four of the 39 stable strains were positive for WNV and for USUV. No association was detected between WNV and USUV positivity at the stable level, although the statistical power was lower than that at the individual level.

## Discussion

Over the last decade, France has witnessed a changing epidemiology of WNV, USUV and TBEV orthoflaviviruses, with an increased incidence of diseases in vertebrate hosts [3, 16, 36].

From cohorts of equines located in the three defined areas of Gironde, we demonstrated WNV seroprevalence rates ranging from 9% (95% CI [6–13]) in the Confluence area to 3% (95% CI [1–8]) in the intermediate area and 1% (95% CI [0–7]) in the Arcachon Basin. These findings are comparable to those of previous reports from Kosovo [37], Spain [38–41], Germany [42–45] and France [18, 19]. In the Camargue region, which has been considered the historical area of WNV circulation in France since the 1960s, the seroprevalence rate among equines estimated between 2016 and 2020 is slightly higher, at approximately 13% [19].

The highest USUV seroprevalence rate observed in horses was obtained in the Confluence area (5%). It was null near the Arcachon Basin.

Therefore, the Confluence area appears to be a local amplification zone for *Culex-borne* orthoflavivirus circulation. Our data revealed a low percentage of seropositive individuals infected with TBEV (0.3%). Nevertheless, this low seroprevalence rate does not allow us to conclude that TBEV is circulating in south-western France. The virus is known to circulate in the north-eastern part of the country [46–48], with outbreaks detected further south in Jura and other regions [49]. Further investigations in ticks and reservoir animals, such as rodents, are needed to confirm the presence of TBEV in the Nouvelle Aquitaine region.

In this study, we analysed the risk factors that might affect the exposure of horses to WNV infection in the Confluence zone. The risk of exposure to WNV increased when horses were kept outdoors and thus were more exposed to vector bites and when the stable was close to the northernmost special protected areas (SPAs) in the Confluence zone. SPAs were created to preserve the biodiversity they harbor. In and around these areas, bird and vector species assembly may favour local WNV circulation, as already suggested in south-eastern France. This finding is in line with the association, at the European level, between West Nile disease occurrence in humans and the local diversity of wild bird species [50, 51].

Age, sex, horse color and distance to the nearest water surface were not identified as risk factors associated with WNV and USUV seropositivity. Our data are consistent with those of García-Bocanegra et al. [52] and Vanhomwegen et al. [53], who reported no significant differences among age classes; Durand et al. [18] in France; Hassine et al. in Jordania [54]; and Bażanów et al. in horses in Poland [55], who reported the absence of an effect of sex on WNV and USUV seropositivity. Analyses of the color of horse coats are rare in WNV seroprevalence studies in horses. The results from several research groups are disparate, with studies showing an association between darker color and a lower WNV seroprevalence rate [56, 57] and others demonstrating no correlation between this variable and WNV and USUV seropositivity [58].

Interestingly, we did not find a correlation between the seroprevalence of mosquito-borne viruses and the nuisance caused by mosquitoes evaluated by owners. As it is a personal assessment and may vary from one individual to another, our data here do not allow conclusions to be drawn.

The number of equine and avian cases recorded by the French National Reference Laboratory for West Nile during the 2023 transmission season suggests that the virus tends to establish itself and extend its range [59]. Cases have been detected in départements bordering the Gironde, especially in Charente-Maritime and Charente.

Carrying out seroprevalence surveys such as the one conducted in this study is essential for strengthening monitoring of WNV circulation. This helps us understand viral emergence and evaluate the intensity of circulation in new areas. The seroprevalence survey carried out close to syndromic cases diagnosed in 2022 identified areas with high viral circulation. In 2023, these data enabled mosquito traps to be set in mid-July, providing rapid evidence of active circulation of the West Nile and Usutu viruses in the Confluence area. These data have played a major part in the rapid implementation of the measures needed to ensure the safety of products derived from the human body (blood and organ donations) throughout Gironde and Charente Maritime, a neighbouring département [60]. Horse owners located in the Confluence zone were encouraged to vaccinate their animals to prevent WNV infection in 2024 and the development of severe forms of the disease.

This first seroprevalence study conducted in equids located in Gironde on the Atlantic coast of France revealed that WNV, USUV and an orthoflavivirus belonging to the TBEV serocomplex circulated in this area and infected horses in 2022. Horses, known as sentinels of WNV infection, exhibited intense circulation of this virus in this region of France as well as equine USUV-specific infection. This study confirmed the usefulness of serosurveys in horses for detecting the circulation of orthoflaviviruses, especially WNV, in specific regions.

## Supplementary Information

**Additional file 1. Mixed effects logistic regression model of the VNT WNV serological status of the horses sampled in the confluence zone, including all the independent variables.**

## Data Availability

The datasets used and/or analysed during the current study are available from the corresponding author on reasonable request.

## Abbreviations

CPE: cytopathic effect
ELISA: enzyme-like immunosorbent assay
IgG: immunoglobulin G
IgM: immunoglobulin M
OR: odd ratio
SPA: special protection area
TBEV: tick-borne encephalitis virus
TCID_50_: tissue culture infectious dose 50
USUV: Usutu virus
VNT: virus neutralization test
WNV: West Nile virus
