# Supplementary material for: Seroprevalence of West Nile, Usutu and tick-borne encephalitis viruses in equids from south-western France in 2023
Source: Vet Res. 2025 Apr 24;56:91. doi: 10.1186/s13567-025-01508-w (PMC12023385; doi:10.1186/s13567-025-01508-w)
Supplement: Supplementary file 1 — Additional file 1. Mixed effects logistic regression model of the VNT WNV serological status of the horses sampled in the confluence zone, including all the independent variables. [file 13567_2025_1508_MOESM1_ESM.docx]

**Additional file 1 Mixed effects logistic regression model of the VNT WNV serological status of the horses sampled in the Confluence zone, including all the independent variables.**

| **Variable** | **Value** | ***p*-value** | **Odds-ratio (95% CI^b^)** |
| --- | --- | --- | --- |
| Intercept |  | 0.20 | 0.05 (0.00-8.22) |
| Type of housing | Pastures and indoors^a^ |  |  |
|  | Pastures only | 0.05 | 3.66 (1.06-14.54) |
| Distance to the Northern SPA | Increase by 1 km | 0.68 | 1.03 (0.87-1.21) |
| Distance to the Southern SPA | Increase by 1 km | 0.44 | 0.94 (0.79-1.09) |
| Distance to nearest water body | Increase by 1 km | 0.20 | 0.72 (0.40-1.19) |
| Sex | Female^a^ |  |  |
|  | Male | 0.11 | 2.20 (0.87-6.03) |
| Hair colour | Light^a^ |  |  |
|  | Dark | 0.16 | 0.52 (0.21-1.31) |
| Age | Increase by 1 year | 0.26 | 1.04 (0.97-1.11) |

^a^ Reference class for the calculation of the odds-ratio. ^b^ Confidence interval
